# Supplementary material for: Dual Stimuli-Responsive Multifunctional Silicon Nanocarriers for Specifically Targeting Mitochondria in Human Cancer Cells
Source: Pharmaceutics. 2022 Apr 13;14(4):858. doi: 10.3390/pharmaceutics14040858 (PMC9028052; doi:10.3390/pharmaceutics14040858)
Supplement: Supplementary file 1 [file pharmaceutics-14-00858-s001.zip › pharmaceutics-1660947-supplementary.pdf]

pharmaceutics

Supplementary Material

Dual Stimuli–Responsive Multifunctional Silicon Nanocarriers for Specifically Targeting Mitochondria in Human Cancer Cells

Vy Anh Tran [1,†], Giau Van Vo [2,3,†], Mario A. Tan [4], Joon-Seo Park [5], Seong Soo A. An [6,*] and Sang-Wha Lee [1,*]

1. Instrumental Analysis

***Scanning electron microscopy (SEM)*:** Hitachi S-4700 FE-SEM (Japan) was used to measure surface morphology of as-prepared samples at 15 kV of accelerating voltage. The sample solution was treated in an ultrasonic bath (for 30 s) and vortex mixer (5 s) and the treated sample was dropped on the cover glass (22 × 22 mm). After drying, the cover glass pieces were attached to the polished aluminum stubs substrate using double-sided carbon tape. Before SEM analysis, the samples were coated with gold or palladium for more accurate size measurements.

***Transmission electron microscopy (TEM)*:** TEM specimens were prepared by placing one drop (10 μL) of the nanoparticle solution onto a carbon-coated copper grid and drying at room temperature. All sample solutions were treated in an ultrasonic bath (30 s) and vortex mixer (5 s) to reduce aggregation of nanoparticles before the deposition process. Low-resolution TEM was performed with a Hitachi H-7600 (Japan) microscope operating at 80 kV, and high-resolution TEM was performed with a Tecnai G2 F30 (Germany) microscope operating at 200 kV.

Fourier Transform Infrared Spectra (FTIR): After powder samples were dried by a freeze-dryer, a small amount of the powder was directly put between two support plates without hygroscopic material (such as NaCl or KBr). The FTIR spectra were recorded using a FTIR spectrometer (Vertex 70, Bruker, USA).

***UV–visible absorption*:** In vitro release tests were performed to determine the released amounts of drug from mesoporous silicon nanocarriers. Immediately after drug-loaded nanocarriers were added into the PBS solution, an aliquot was periodically sampled from the solution to monitor the cumulative amounts of released drug by UV–vis spectroscopy (NanoDrop; NanoDrop Technologies, Wilmington, DE, USA).

***Raman spectroscopy*:** Raman spectra of the samples were measured by a micro-Raman spectrometer (ANDOR Monora 500i) equipped with light microscope BX43-Olympus. Indium tin oxide (ITO) glass was used as the supporting substrate to check Raman spectra of the samples. First, ITO glass was washed by sonication for 5 min in HPLC-grade water, and then sample solution was repeatedly deposited on the ITO glass. After completing the deposition process, dropped samples were dried at room temperature and analyzed by Raman spectroscopy.

The Yield of Nanomaterial Synthesis

❖ *Synthesis of silica nanoparticle (MSN)*

The yield of the SiO_2 was calculated from the weight of the collected after the steps of synthesis versus the theoretical weight of SiO_2 obtained from TEOS.

TEOS (Molar mass: 208.33 g·mol^{-1}, Density: 0.933 g/mL at 20 °C).

SiO_2 (Molar mass: 60.084 g·mol^{-1}).

Theoretically, 10 mL of TEOs will produce 2.69 mg of SiO_2. In practice, SiO_2 was synthesized by a sol–gel reaction and the final fabrication of nanocarriers has an efficiency of 71.4% (1.92 mg/2.69 mg).

❖ *Synthesis of silicon nanoparticle (MSN into MPSi)*

Theoretically 600 mg of SiO_2 will produce 280 mg of Si. Actual experimental performance achieved 48.5% yield (136 mg/280 mg).

The synthesized FMPSi-Cis@GO@DQA proved to be efficient in inhibiting the growth of two cancer cell lines SH-SY5Y (IC_{50} = 29.2 µg/mL) and HeLa (IC_{50} = 24.4 µg/mL), while non-toxic to the normal HEK-293 cells (IC_{50} > 100 µg/mL).

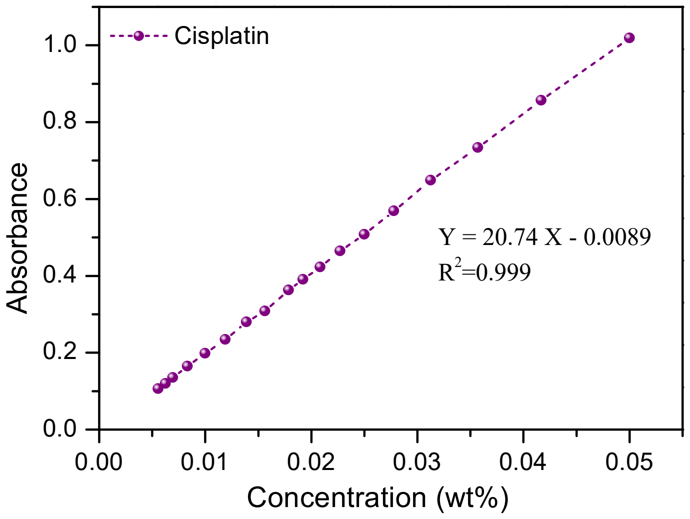

Figure S1. Linear part of the standard curve of cisplatin absorbance at 301 nm in PBS over the concentration range (0.005–0.05 wt%).

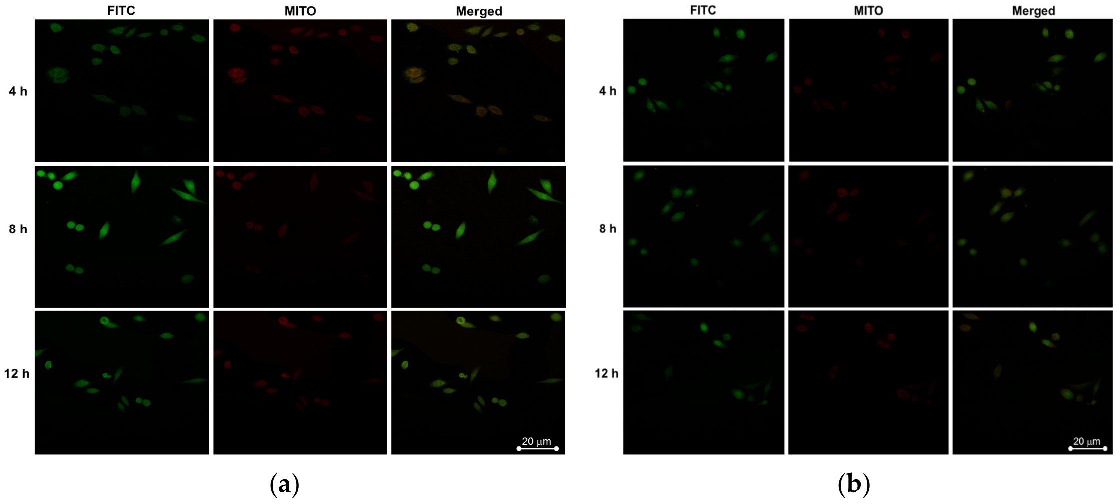

Figure S2. Confocal microscopy images of (**a**) HeLa cells treated with 25 µg/mL FMPSi for 4 h, 8 h, and 12 h incubations, (**b**) HeLa cells treated with 25 µg/mL FMPSi-Cis for 4 h, 8 h, and 12 h incubations.

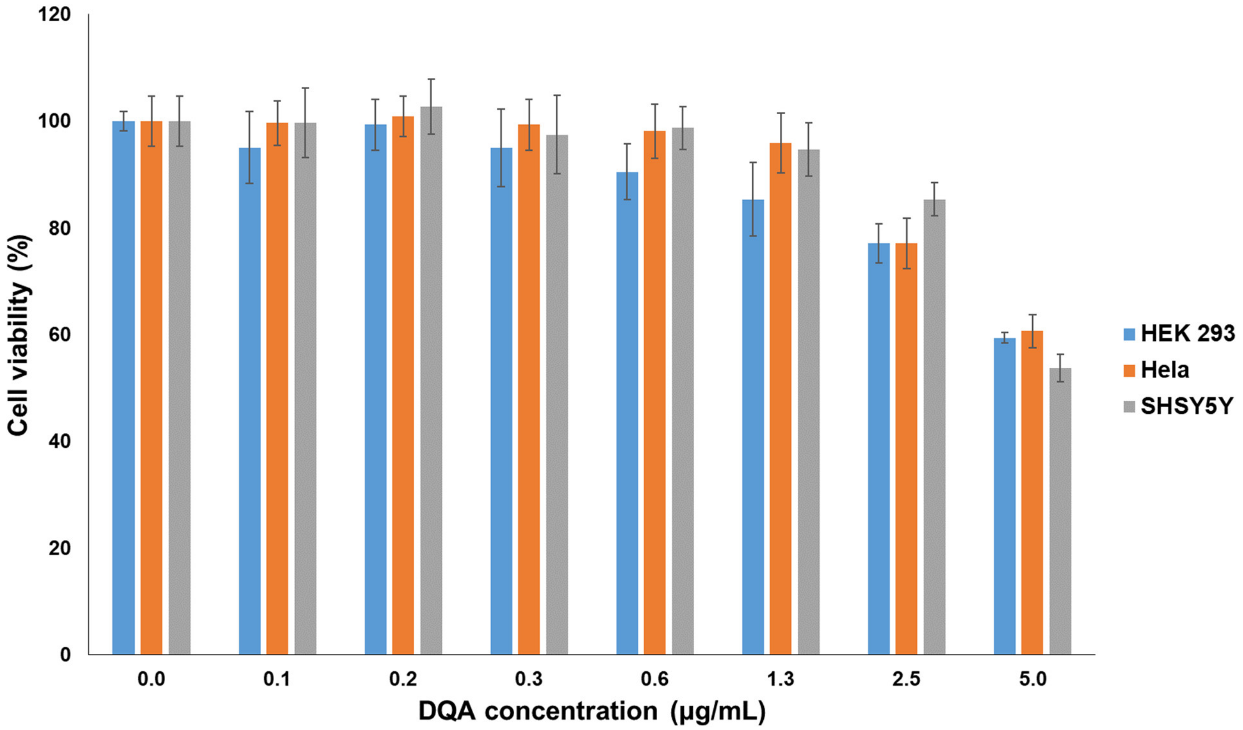

Figure S3. Cytotoxicity test of DQA: Cell viability of HEK293, Hela, and SH-S5Y5 cells after the treatment of different concentrations DQA for 48 h. Data are represented as mean ± SD (n = 4).

Table S1. Zeta potentials of as-prepared samples at neutral pH.

Samples	MPSi	MPSi-OH	MPSi @APTMS	MPSi @APTMS-FITC (FMPSi)	FMPSi@GO
Zeta potentials	+0.90 ± 0.48	−8.16 ± 2.23	+3.85 ± 1.70	−14.95 ± 2.04	−34.80 ± 3.52

The data were expressed as the means ± the standard deviations (SD). The mean value (μ_x) and standard deviation (SD) of release data were calculated by the following formula of (S1) and (S2).

$$\mu_x = \frac{\sum_{i=1}^{n} x_i}{n} \tag{S1}$$

$$\mathrm{SD} = \sqrt{\frac{\sum_{i=1}^{n}(x_i - \mu_x)^2}{n-1}} \tag{S2}$$

Table S2. BET–BJH properties of MSN, MPSi, and FMPSi samples.

As-Synthesized Particles	BET–BJH Method	
	Surface Area (1) (m²/g)	Pore size (2) (cm³/g)
MSN	1407	2.78
MPSi	427	2.52
FMPSi	807	3.92

(1) The surface area was estimated according to the Langmuir Surface Area method. (2) The pore size and pore volume were calculated by the Barrett–Joyner–Halenda (BJH) method.

Table S3. The summary of fitted parameter values of kinetic models: cisplatin release from MPSi-Cis at pH 5.5 and pH 7.4; cisplatin release from FMPSi-Cis@GO at pH 5.5.

Drug-Loaded Nanocarriers	Diffusion Models	Equations	Fitted Parameters
MPSi-Cis (at pH 5.5)	Fick's law	$Q_t/Q_\infty = 1\text{-}Ae^{-k_F t}$	$k_F = 0.44$ (A = 1)
MPSi-Cis (pH 7.4)	Fick's law	$Q_t/Q_\infty = 1\text{-}Ae^{-k_F t}$	$k_F = 0.68$ (A = 1)
FMPSi-Cis@GO (pH 5.5)	K-P model	$Q_t/Q_\infty = k_R t^n$	$k_R = 12$, n = 0.69

K-P model indicates the Korsmeyer–Peppas model.

Table S4. Loading and release amounts of cisplatin from FMPSi@GO at different conditions (pH and NIR) and cumulative release fractions.

**Material**	(a) **Cis Loading Amounts of in DMSO (mg cis/mg Material)**	(b) **Cis Release Amounts in DMSO (mg cis/mg Material);**	(c) **Cis Loss (%)**	(d) **Cis Release Amounts *at pH 5.5 and no NIR* (mg cis/mg Material); Q_t/Q_o(%)**	(e) **Cis Release Amounts *at pH 7.4 and no NIR* (mg cis/mg Material); Qt/Q_o(%)**	(f) **Cis Release Amounts *at pH 5.5 and NIR* (mg cis/mg Material); Q_t/Q_o (%)**	(g) **Cis Release Amounts *at pH 7.4 and NIR* (mg cis/mg Material); Q_t/Q_o (%)**
FMPSi@GO	0.4072	0.3162	22.3%	0.1584; 50.1%	0.0784; 24.8%	0.2463; 77.9%	0.2394; 75.7%

Cis = Cisplatin; PBS = Phosphate Buffer Solution; and DMSO = Dimethyl Sulfoxide. (a) Initial loading amounts of cisplatin by FMPSi and FMPSi@GO. (b) The cisplatin release amounts in DMSO. This amount can be considered as the initial loading amounts of cisplatin, Q_o, because some drug loss should occur during the surface modification. (c) Drug loss (%) by the surface modification process can be estimated as the ratio (×100%) of (b) to (a). (d–g) Release amounts of cisplatin, Q_∞, from the in vitro release test in PBS at different conditions (pH and NIR) and cumulative release fractions (%).
